# Person-centered medical interview

**DOI:** 10.3325/cmj.2012.53.310

**Published:** 2012-08

**Authors:** Veljko Đorđević, Marijana Braš, Lovorka Brajković

**Affiliations:** Center for Palliative Medicine, Medical Ethics and Communication Skills, University of Zagreb School of Medicine, Zagreb, Croatia mbras@kbc-zagreb.hr

## Abstract

**Abstract:**

We are witnessing an unprecedented development of medical science and personalized medicine. However, technological superiority must not make us lose sight of the physical, psychological, social, and spiritual totality of the patient. The core of the medical profession has always been and will be the relationship between the health professional and the person seeking assistance. However, the traditional relationship between the physician and the patient has changed and is greatly impacted by huge social, philosophical, economic, and scientific developments. It is important to develop and promote the culture of health instead of the culture of illness through a patient-doctor collaborative partnership, as well as partnership among professionals. Person-centered medical interview is an important bridge between personalized and person-centered medicine.

We are witnessing accelerating advances in medical science that promise to revolutionize health care and significantly improve patients’ health outcomes. Personalized medicine is an innovative approach originating from life sciences, based on each person’s unique genetic, clinical, and environmental information (1). The nature of diseases (onset, course, and outcome) is as individual as the people who have them. Personalized medicine is about making the treatment as individualized as the disease by identifying the information that allows making accurate predictions about a person’s susceptibility to disease, the course of disease, and its response to treatment (1). It is a technology of the future, but it is already having an impact on the way patients are being treated. However, the adoption of personalized medicine should be driven by patients’ needs and not the science itself. Science is moving much faster than medical practice at this point. Personalized medicine has the potential to fundamentally change the way health care is practiced and delivered. However, its success will depend on the ability of pharmaceutical companies, health professionals, medical educators, insurance companies, and policy makers to collaborate, and to create an integrated framework that meets the health care needs of the people. We must not forget that the most important part of personalized medicine is the patient, the person. Physicians are offered a powerful ability to match patients to specific highly targeted therapies and to maximize their chances for a successful treatment. But, do we treat patients as persons or merely as objects with a disease? How to refocus the medicine and health on the person in its entirety? How can we use biomedical views and not forget the patient’s own view on his or her condition and the context in which the disease develops, while taking into account the most recent scientific knowledge?

The answer could be in person-centered medicine, a movement which is growing toward the formulation of a medicine *of* the person (of the totality of the person’s health, including the ill and positive aspects), *for* the person (to assist the fulfillment of the person’s life project), *by* the person (with clinicians as full human beings, professionally competent, and with high ethical aspirations), and *with* the person (in respectful collaboration with the person who is doing the consulting) (2). The extension of the person-centered care initiative took place five years ago through the annual Geneva Conferences on Person-centered Medicine organized in collaboration with the World Medical Association, the World Health Organization (WHO), the International Council of Nurses, the International Alliance of Patients’ Organizations, and twenty other international medical and health institutions. The Geneva Conferences process led to the emergence of the International Network (recently renamed College) of Person-centered Medicine. Among the prominent developments from these institutional efforts are a recent research project, in collaboration with the WHO, on the systematic conceptualization and measurement of person- and people-centered care, and the design of new clinical procedures such as the Person-centered Integrative Diagnosis model and guide. On top of this, the *International Journal of Person Centered Medicine* was launched in 2011 as a joint venture of the International College of Person-centered Medicine and the University of Buckingham Press in the United Kingdom (2,3).

In both personalized medicine and person-centered medicine initiatives, the emphasis is on the person. However, personalized medicine is more focused on science and person-centered medicine on holistic and humanistic approach.

## Where is the link between personalized and person-centered medicine?

As health care in general is a very complex system, reflecting social changes, in recent decades great attention has been paid to the quality of communication in medicine. Communication is the most widely used clinical skill in medical practice, which includes all participants of the health system. There are a lot of types of communication, to mention only a few: communication between the patient and physician, between the patient and other members of the health care team (nurses, psychologists, teachers, social workers, medical technicians, physical therapists, etc), between physician/health care professionals and patients’ family members, between members of medical teams and multidisciplinary teams, among health professionals within professional associations, between physicians and local and national governments and intergovernmental organizations, civil society organizations, insurance companies, pharmaceutical companies, the media, etc. Training in communication skills in medicine is essential for a long-term theoretical, practical, individual, and team work.

Patients have the need to feel known and understood; such needs are also referred to as affective and instrumental needs. Fulfillment of these needs depend on communication skills – patient’s skills and physician’s skills, which help to bridge the distance between people in an interaction. Communication is an integral part of any relationship with patients and their families, and represents the key to the success of a medical team. Communication and relationships have an impact on patients’ experience of care, improve patients’ adherence to treatment regimens, clinical outcomes, quality of care, and patients’ safety, contribute to teamwork and cultural sensitivity, and reduce medical malpractice risk (4,5). Experience and talent are not enough to ensure optimal communication. There are those who are more or less talented, more skilled or less skilled in communication, but it is encouraging that communication is a skill, which can be taught. Today it is more than ever important to balance the humanistic approach and medical sciences because every consultation is a unique process, in which a physician should see not only the problem presented by the patient, but also the healthy parts of the patient’s personality. In the patient-physician (health professional) relationship, it is important to focus on stimulating healthy and creative forces in our patients that are so important for their coping with the disease, maintaining hope, fighting for life, and not giving up. Do we help our patients by focusing only on their disease or do we accept them in their entirety, recognizing their life goals, and seeing them in the context of continued growth and development? A patient’s experience of illness, knowledge, and the nature of the illness are all intertwined in medical decisions and outcomes. An understanding of the mind-body relationship must be appreciated as well. An essential component of medicine is caring for the patient. It seems that as the physician’s ability to cure the disease has increased, the capacity for caring has lessened, although sometimes caring is more important than curing. We should accept the person seeking help as a whole and that person should also accept us as persons who provide help and can be trusted. An ill person and a health care professional walk together and grow together. The treatment process exists only while the trusting relationship exists. Due to the recent advances in neuroscience, we are now able to describe and discuss the biological mechanisms that underlie the health professional-patient relationship. We now know that different physiological and biochemical mechanisms take part in complex functions, like trust, hope, empathy, and compassion, which are all very important elements in the health professional-patient relationship. The main advantage of a neuroscientific perspective of the health professional-patient relationship is that doctors, psychologists, and other health professionals can better understand the kind of changes they can induce in their patients’ brains, further boosting the professional’s empathic and compassionate behavior (6,7).

Only good communication can provide and establish good relationship between the health professional and patient, and the most important aspect of communication is medical interview, as a bridge from bench to bedside to community.

The medical interview provides a framework for exploring and understanding patients’ concerns, fears, misconceptions, and what they bring to their illness while taking into consideration their culture, availability of treatment options, and financial considerations (8). Medical interview is a complex process of obtaining information for the purpose of making a diagnosis and it is an extremely important factor in establishing the relationship between health professionals and patients (8,9). The essential elements of the integrated patient-centered and physician-centered interview are to build a relationship, open the discussion, gather information, understand the patient’s perspective, share information, reach agreement, and provide closure. Physician-centered interview includes asking specific questions in order to establish the diagnosis. Most of the conversation is done by the physician. This approach in most cases ignores important personal information about the patient, his or her personality, and emotions (10,11). On the other hand, patient-centered interview is focused on understanding the patient’s perceptions of illness. Patient-centered communication style aims to identify the patient’s needs. Likewise, the patient’s open and clear presentation of his or her reasons for visit adds to an effective and efficient encounter, which makes the patient feel helped, empowered, and cared for (10,11). As a consequence of the differences in background and expertise, the patient’s perspectives often clash with the perspectives of the physician (12). Integrating patient-centered interviewing with physician-centered interviewing results in the most complete, accurate, and diagnostically powerful data set – the patient’s biopsychosocial story, and strengthens the patient-physician relationship (10). Physicians with effective relationship skills will have more satisfied patients, they will better cope with emotionally troubling situations, be able to give emotionally more to patients and, in turn, they will get more satisfying responses from them (11). Physicians’ comprehensive knowledge of patients and patients’ trust in their physician are the variables most strongly associated with adherence, and trust is the variable most strongly associated with patients’ satisfaction with their physician (13). Attentiveness and worth, empathy, respect, support, and partnership are basic relationship skills that help build physician-patient rapport (14). Attentiveness to the patient as a person shown by verbal and non-verbal behaviors is a prerequisite of all relationship building. Closely related to attentiveness is empathy, which refers to the physician’s ability to enter into the patient’s world and see things with their eyes. It is very important to be aware that every patient has a different perspective. Everyone interprets the world against his/her own background of experience (14). Building relationships is extremely important, not only in the beginning, but during the whole patient-health professional interaction (14). Many studies indicate that more information provided by the physician about the disease will reduce psychological stress, alleviate the symptoms, and improve the outcome of treatment (15). On the other hand, if the physician provides only written information about the disease and the diagnosis, patients often respond with bad mood and anxiety (15,16). Reaching agreement is a complex process that begins with identifying the nature of the problem, and includes gathering and sharing information about biomedical knowledge, feelings, concerns, and preferences, toward building consensus and collaborating to create shared decisions (17). In ''paternalistic'' model, decisions are made by physician without patients’ involvement (18). In the “collaborative” model, the physician and patient share information and thoughts, as well as decisions about diagnostic and therapeutic procedures and plans (18). Psychological and physical health and efficiency of care improve and frequency of malpractice claims is reduced when patients are more involved in making decisions about their care.

Focused interventions, either with patients or their clinicians, enhance patients’ involvement in decision-making (16,17). Communication skill training is now internationally accepted as an essential component of medical education (19). However, learners and teachers of communication skills continue to experience problems in integrating communication with other clinical skills. One factor contributing to these problems is that learners confront two apparently conflicting models of the medical interview: a communication model describing the process of the interview and the “traditional medical history” describing the content of the interview (19). Kurtz et al propose a comprehensive clinical method that explicitly integrates traditional clinical method with effective communication skills (Calgary-Cambridge guides, 19). They devised a content guide for medical interviewing that is more closely aligned with the skills used in communication training, and incorporates patient-centered medicine into both process and content aspects of the medical interview (19).

Although Calgary Cambridge is a widely accepted form of medical interview, it does not encompass enough questions about the positive aspects of the patient – what is healthy, which are the positive aspects of personality, what kind of positive coping mechanism the person uses, etc. This approach is more focused on the disease rather than an individual’s health and it does not observe the individual in his/her entirety. In this way, it ignores questioning the quality of life and satisfaction with life. Therefore, the Zagreb model of Person-centered medical interview is currently under development by the authors (Veljko Đorđević, Marijana Braš, and Lovorka Brajković). The Zagreb model is focused not only on the disease or illness but on patient’s quality of life in the context of health and disease. This model is currently in the process of validation; it is used for the patients with cancer and patients with chronic pain syndrome. The obtained data will be a starting point for the implementation of the Zagreb person-centered medical interview model for all patients.

## Conclusion

Although personalized medicine is more focused on science and person-centered medicine is more focused on holistic and humanistic approach, it does not mean that they are mutually opposed. On the contrary, there is a strong link between them. Person-centered medicine has emerged as a response to the organ-specific, technical, fragmented medical treatment and care, and its main component is the cornerstone for successful treatment and care (14). It is very important for health professionals to use their communication skills to provide successful medical treatment and care, to establish and build good relationship with their patients, and to be aware of uniqueness of every patient. Health professionals must adhere to many of the principles of evidence-based medicine, but not forget to use person-oriented and person-centered approach. Human relationship is what matters most!
